# Almonds ameliorate glycemic control in Chinese patients with better controlled type 2 diabetes: a randomized, crossover, controlled feeding trial

**DOI:** 10.1186/s12986-017-0205-3

**Published:** 2017-08-02

**Authors:** Chiao-Ming Chen, Jen-Fang Liu, Sing-Chung Li, Chen-Ling Huang, An-Tsz Hsirh, Shuen-Fu Weng, Mei-Ling Chang, Hung-Ta Li, Emily Mohn, C-Y. Oliver Chen

**Affiliations:** 1 Department of Food Science, Nutrition, and Nutraceutical Biotechnology, Shih-Chien University, No.70, Dazhi St., Zhongshan Dist, Taipei City, 104 Taiwan; 2 Department Nutrition & Health Sciences, Chang Gung University of Science and Technology, No.261, Wenhua 1st Rd., Guishan Dist, Taoyuan City, 33303 Taiwan; 3 School of Nutrition and Health Science, Taipei Medical University, 250 Wuxing St, Taipei City, 110 Taiwan; 4 Division of Endocrinology and Metabolism, Department of Internal Medicine, Taipei Medical University Hospital, 252 Wuxing St, Taipei City, 110 Taiwan; 5 Division of Endocrinology & Metabolism, Department of Internal Medicine, Taipei Medical University-Shuang Ho Hospital, No.291, Zhongzheng Rd., Zhonghe District, New Taipei City, 235 Taiwan; 6 Antioxidants Research Laboratory, Jean Mayer USDA Human Nutrition Research Center on Aging, Tufts University, 711 Washington St., Boston, Massachusetts 02111 USA

**Keywords:** Almonds, Diabetes mellitus, HbA1c, Inflammation, Oxidative stress

## Abstract

**Background:**

Almonds can decrease glycemic index of co-consumed foods and are a rich source for oleic acid and α-tocopherol. The aim of the randomized, crossover, controlled feeding trial was to examine whether as compared to NCEP step II diet as control (CON), ~60 g/d almonds (ALM) added to CON would improve glucoregulation and cardiovascular disease (CVD) risk factors in 33 Chinese T2DM patients.

**Methods:**

Forty T2DM patients were enrolled and randomly assigned to receive CON or ALM for 12 wks after a 2-wk. run-in period. Blood and urine samples were collected in the beginning and at the end of each dietary intervention phase for the assessment of biomarkers of glucoregulation, lipid profile, inflammation, and oxidative stress.

**Results:**

While ALM had a better overall nutritional quality than CON, neither ALM nor CON improved the glycemic status as the primary study outcome and other CVD risk factors, except the circulating nitric oxide being decreased by ALM compared to CON. Among 27 of 33 patients with the baseline HbA1c ≤8, ALM decreased post-interventional fasting serum glucose and HbA1c by 5.9% and 3.0% as compared to that of CON, respectively (*P* = 0.01 and 0.04). Mean total and LDL-cholesterol concentrations were not changed by both diets.

**Conclusions:**

These results suggest almonds incorporated into healthful diets can improve glycemic status in diabetic patients with a better glycemic control.

**Trial registration:**

NCT01656850, registered 13 January 2012.

## Background

Diabetes is associated with the development and progression of cardiometabolic diseases [1]. According to the 2015 International Diabetes Federation report, the prevalence of diabetes in adults was 8.8% (415 million people) globally, with 85–90% being type 2 diabetes mellitus (T2DM) [2]. The percentage was predicted to increase to 10% by 2035, mainly due to the epidemics of overweight/obesity [3]. Cardiovascular disease (CVD) is the most common cause of morbidity and mortality for patients with the diabetes, underscoring the critical need for proper management of CVD risk factors [4]. Oral hypoglycemic medications are the first line of the diabetic management, but lifestyle modifications, including diet and physical activity, are the cornerstone to help maintain/improve the diabetic condition and protect against more severe complications [5, 6].

Patients with T2DM are generally instructed by medical professionals to follow a nutrition therapy of their own preference, in which monitoring consumption of carbohydrate-rich foods and increasing fruits, legumes, vegetables, whole grains, and dairy products are generally recommended to control wide blood glucose swings [7]. Though tree nuts as a food group have not been included in any specific nutrition recommendations [7], they can be considered in nutrition therapy(s) [8] because of their capability to decrease glycemic index of co-consumed foods and to improve lipid profiles and biomarkers of inflammation and oxidative stress [9–18]. However, a small body of clinical evidence illustrating the effect of almonds on blood glucose control in patients with T2DM is mixed. We and others [16, 19] found that almonds improved blood glucose control in T2DM patients, but null outcomes were also noted in Lovejoy et al. study [19]. Though mechanisms contributing to the mixed results remain to be elucidated, they can be attributed to the divergences in study design (dose, timing and means of almond consumption, duration, controlled feeding) and study participants (diabetes history, medication use, glycemic management, ethnicity, diet, CVD risk factors).

Our previous 4-week controlled feeding study with Chinese patients with T2DM and mild hypercholesterolemia [16] showed that almonds (~60 g/d) added to replace 20% calories of a National Cholesterol Education Program step II diet (NCEP II) [20] improved glycemic control, lipid profile, oxidative stress status, and inflammation as compared to the NCEP step II diet. In addition, we noted in this study that almonds decreased body fat, an improvement associating favorably with insulin resistance, inflammation, and other CVD risk factors [10, 21–23]. Because of the relatively short study duration and small sample size, a more robust trial is needed to substantiate the health benefits of almonds in glycemic status of patients with T2DM. Thus, we conducted a longer clinical trial to examine the effect of almonds incorporated to replace 20% calories (~60 g/d) of the NCEP II diet on glucoregulation, lipid profile, inflammation, and oxidative stress in Chinese patients with T2DM. The primary outcome of the study was HOMA-IR and other outcome measures included fasting HbA1c, C-peptide, high sensitive C-reactive protein, E-selectin, ICAM-1, endothelin-1, nitric oxide, oxidized LDL, protein carbonyl in plasma or serum, postprandial glucose and insulin responses to a standard breakfast, and urinary isoprostanes. However, mild hypercholesterolemia was not an inclusion criterion because it has become a standard treatment in Taiwan when patients with T2DM diagnosed with hypercholesterolemia have been generally prescribed with lipid-lowering drugs such as statins.

## Methods

### Subjects

Patients previously diagnosed with T2DM by an attending physician of the Endocrine Clinic of either the Taipei Medical University Hospital (TMUH) or the Taipei Medical University-Shuang-Ho Hospital in Taiwan were recruited for the trial. The inclusion criteria included age between 40 and 70 y, BMI: 24 to 35 kg/m^2^, HbA1c: 6.5–10%, and regular use of prescribed oral hypoglycemic agents [sulfonylureas (e.g., Glibenclamide), biguanide (e.g., metformin), DPP-4 inhibitor (e.g., Januvia), and α-glucosidase inhibitor (e.g., acarbose)]. The exclusion criteria included regular use of insulin, oral steroids or anti-inflammatory agents, ≥5% body weight change in the last 6 mo, diagnosed CVD, stroke, gastrointestinal diseases, inflammatory bowel disease, chronic kidney disease, hepatobiliary disease, renovascular disease, endocrine diseases, hyperuricemia, autoimmune diseases, active treatment for cancer of any type ≤1 y, poor hypertension control (systolic blood pressure ≥ 150 mmHg and/or diastolic blood pressure ≥ 95 mmHg), known allergies to nuts of any kind, frequent nut consumption (≥3 oz./wk), regular use of any dietary supplements or homeopathic remedies, daily ethanol intake of ≥2 drinks and smoking. Due to insulin dosage is normally adjusted with carbohydrate intake, there might be a safety concern with the decrease of carbohydrate intake from 56% to 47% when fat-rich almonds were incorporated into study diet. Thus, patients using insulin were excluded from the study.

After enrollment, subjects were asked to adhere with medication regimens and to refrain from consuming nuts during the 2-wk. run-in period and during the intervention. The study protocol was approved by the Institutional Review Board of the Taipei Medical University, and written informed consent was obtained from participants before study procedures were performed. The study was registered at ClinicalTrials.gov (no. NCT01656850).

### Study design

**Fig. 1:**
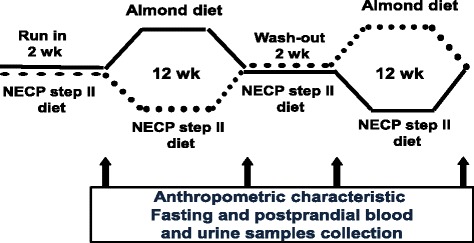
Study design.

The study was a 28-wk. randomized, crossover, controlled feeding trial with a 2-wk. run-in period and a 2-wk. washout between alternative diets. The study duration was selected because it was required for the assessment of HbA1c, which is indicative of the long-term glycemic status. The enrolled patients were randomly assigned to receive one of the study diets for 12 wks after the run-in period, during which they consumed CON (Fig. 1). A randomization scheme was prepared by a biostatistician using a standardized computer program for two treatment groups in a crossover design. ALM and CON were prepared by the metabolic kitchen of the TMUH. A total of 4 overnight fasting blood and urine samples and anthropometric data were collected from each participant during the trial. At the beginning and the end of each intervention phase, Postprandial glycemic response to a standard breakfast comprising 2 slices of white bread and 240 mL unsweetened soy milk (containing 45 g carbohydrate, 15 g protein, and 8 g fat) was also assessed, and blood was collected prior to and at 30, 60, 90, 120, and 180 min post breakfast. Instead of using the standard 75 g glucose employed in the oral glucose tolerance test, we used standard breakfast to prevent the incidence of hyperglycemia and to provide a control meal for postprandial glycemic response test. The breakfast food items were selected as they were commonly consumed in Taiwan and provided an adequate amount of glucose for the test. The postprandial glycemic response test was performed after subjects were fasted overnight. Area under curve of postprandial glucose or insulin concentration and time course was calculated using the trapezoid rule [24]. All subjects were instructed to maintain their habitual physical activity and intensity throughout the study to minimize any potential influences on blood glucose and lipid.

### Study diets

Study meals were tailored to meet daily energy need of each subject to maintain body weight, calculated using the information of anthropometry and daily physical activity level. Body weight was monitored weekly for the adjustment of daily energy intake in order to maintain BW throughout the whole trial. CON was formulated according to the NCEP II [20] and provided daily calories from carbohydrate, protein, and fat at 55, 17, and 28%, respectively. These percentages were comparable to those generally consumed by Taiwanese. Calories from SFA and PUFA were <7 and 10%, respectively, and cholesterol content were <200 mg. A 2-week menu rotation was used to make meals more appetizing to the participants. ALM was prepared by incorporating roasted, unsalted whole almonds with skins or derived powder to replace 20% calories of CON. The dose was selected based on the positive data of our previous study [16] and the data of Josse et al. (2007) study in which almonds at the dose of 60 and 90 g were effective to lower the glycemic index of white bread in healthy people, but effective at the dose of 30 g [14]. Almonds were either incorporated into entrees or deserts or consumed as a snack. Roasted almonds were used in the trial because their more appealing taste to most consumers. In our previous study [16], almond powder was used to prepare a list of Chinese foods, such as steamed buns, bread, dumpling skin, daikon radish cake, and pizza dough. Providing the daily dose of almonds in a wide range of study foods was aimed to enhance subject compliance. The average daily almond intake was 60 g. The same batch of almonds generously provided by the Almond Board of California were used in the study. During the intervention, subjects were asked to pick up all of their meals and snacks from the TMUH 3 times a week. To monitor the compliance and assess nutrient intakes, subjects were instructed to bring back all unconsumed foods when they returned the TMUH to pick up meals. The unconsumed foods were weighed and recorded. A compliance calendar was also used to monitor the consumption of almond foods. Nutrients intake of CON and ALM were calculated using the Nutritional Chamberlain Line, Nutritionist Edition, version 2002 (E-Kitchen Business Corp, Taiwan).

### Anthropometric measurements

Body height was measured by a height measuring device. Body weight and % fat mass were assessed using an X-SCAN PLUS body composition analyzer by a bioelectrical method (Jawon Medical, Seoul, Korea). The % fat mass was measured up to a 0.1% accuracy. After ≥10 min of rest in a quiet room, blood pressure was measured twice with 10 min rest between measurements, using a FT-500 R automatic blood pressure meter (Jawon Medical). The final blood pressure values are the mean of 2 measurements.

### Blood and urine collections

Fasting and postprandial blood samples were collected from a venous vein of one arm into vacutainers with and without anticoagulant (EDTA or NaF). Plasma and serum were collected after centrifugation at 1400 × g for 10 min at 4 °C, aliquoted, and then stored in −80 °C until analyses. Morning spot urine was also collected at the study sites after overnight fast and stored at −80 °C until analysis.

### Biochemical biomarkers

Serum AST and ALT, creatinine, and magnesium were all determined using a Siemens Advia 1800 automated chemical analyzer (Erlangen, Germany).

Glucose in NaF plasma and lipid profile in serum were determined using a SYNCHRON LX20 Pro clinical chemistry analyzer (Beckman Coulter, Fullerton, CA). Serum insulin was determined by an electrochemiluminescence immunoassay (Roche, Switzerland), and inter-day coefficient of variation (CV) was 8%. Homeostasis model assessment-Insulin Resistance (HOMA-IR) was calculated according to the formula: insulin × glucose/22.5, with insulin expressed as μU/mL and glucose as mmol/L [25]. C-peptide was measured using a chemiluminescent assay (Siemens Centaur, Erlangen, Germany), and the inter-day CV was 10.7%. Apolipoprotein [26] A-1 and Apo B in serum were measured using PEG enhanced immunoturbidimetric assays with the Siemens Advia 1800 analyzer, and inter-day CV was 4.2 and 5.8%, respectively.

High sensitivity C-reactive protein (hsCRP) in serum was determined in TBA-40FR chemistry analyzer using a latex-enhanced turbidimetric immunoassay (Toshiba, Japan), and inter-day CV was 7.2%. Plasma intracellular adhesion molecule-1 (ICAM-1), E-selectin, and endothelin-1 were assessed using human sICAM-1/CD54 Quantikine ELISA kit (R&D Systems, Minneapolis, MN), human E-selectin/CD62E ELISA kit, (R and D Systems) and endothelin-1 Quantikine ELISA kit (R and D Systems), respectively. Serum nitric oxide (NO) was determined by a colorimetric total nitric oxide assay kit (R and D Systems). Intra-day CV for ICAM-1, E-selectin, endothelin-1, and NO was 2.6, 2.2, 2.8, and 2.9%, respectively, and inter-day CV was 3.9, 12.5, 11.1, and 10.0%.

Total F_2α_-isoprostanes in urine were determined using our routine HPLC-GC/MS method [27]. The final value is expressed after adjusted with creatinine. Intra- and inter-day CV for the assay was 4.5 and 9%, respectively. Protein carbonyl in serum was determined by a colorimetric assay kit (Cayman Chemical, Ann Arbor, MI). Circulating oxidized LDL in serum was determined using an ELSIA kit (Mercodia, Uppsala, Sweden). Intra-day CV for protein carbonyl and oxidized LDL was 1.2 and 1.4%, respectively, and inter-day CV was 4 and 3.7%.

Plasma α-tocopherol was determined using a reverse-phase HPLC method, according to Bieri et al. [28]. Briefly, α-tocopherol in plasma was extracted with hexane and then quantified using an HPLC system (Hitachi, Japan) equipped with a LiChroCART C-18 column (4 × 250 nm, 4 μm; Perkin-Elmer, West Lafayette, IN) and a UV/VIS detector (Hitachi, Japan). The concentration in unknown samples was calculated using a standard curve constructed using authentic α-tocopherol. Intra-day and inter-day CV was 7.5 and 11.3%, respectively.

### Statistical analyses

The sample size was calculated using the HOMA-IR data of our previous study [16]. A sample size of 40 subjects was needed to detect the significant difference in HOMA-IR at the α value of 0.05. The power calculation was performed using the PROC POWER and PAIREDMEANS TEST in SAS as the study was a randomized crossover trial. The HOMA-IR value of pairedmeans used in the calculation was 5.19 and 4.63 and pairedstddevs was 2.55 and 2.10, respectively. Results are expressed as mean ± standard deviation [29]. Student’s t-test was performed to assess nutrients between CON and ALM. Paired Student’s t-test was performed to evaluate the difference of the study outcomes between the baseline and at the end of the washout, and the results showed that there were no significant differences between them. A repeated-measures ANOVA analysis was performed to assess the significance between treatments, using PROC GLM with treatment (ALM vs. CON), sequence (ALM-CON vs. CON-ALM), period (1 vs. 2), and subject as independent variables. The effect of sex and its interaction with other independent variables were insignificant; thus, they were excluded from the model. LSMEANS was employed to evaluate the significance in differences between 2 diets. Normality of the outcomes for the ANOVA analysis was evaluated, and log transformations for the skewed outcomes were made to satisfy the distributional assumptions. Significance was considered at *P* ≤ 0.05 (2-tailed). All analyses were performed using SAS 9.3 (SAS Institute, Cary, NC).

## Results

### Participant characteristics

**Fig. 2:**
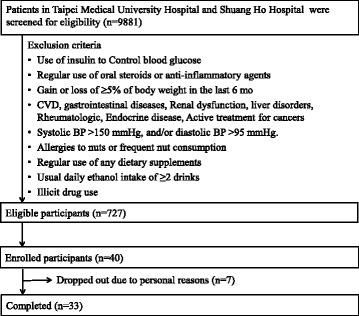
CONSORT chart of the trial.

A total of 40 patients were enrolled and 33 of them completed the whole intervention with full compliance. The compliance to the study protocol was assessed by measuring the amount of unconsumed foods during the trial and checking the compliance calendar of almond consumption (Fig. 2). Two subjects withdrew from the study because of unspecified personal reasons unrelated to the study and 5 withdrew because of constraints related to the controlled feeding, such as meal pick up and irregular working hours. The demographics of 33 completers is illustrated in Table 1. All subjects didn’t change their medication regimen during the whole intervention.

**Table 1:** Demographics of subjects at the enrollment^a^

Attribute	Value
Sex, F/M	20/13
Age, year	54.9 ± 10.5
Diabetic duration, years	4.8 ± 3.2
Use of oral hypoglycemic agent, n (%)	33 (100)
Sulfonylurea, n (%)	17 (52)
Biguanides, n (%)	31 (94)
DPP-4 inhibitor, n (%)	4 (12)
α-glucosidase inhibitor, n (%)	6 (18)
Use of Lipid-lowering drugs, n (%)	8 (24)
Use of Antihypertensive drugs, n (%)	3 (9)

^a^Values are expressed as mean ± SD

### Study diet

Average caloric intake was comparable between CON and ALM phases (Table 2). Subjects consumed 8.7% less calories from carbohydrates and 10.1% more from fats during the ALM phase than the CON phase (*P* ≤ 0.0001). ALM and CON provided 16 and 7% calories from MUFA, respectively. As 60 g almonds provide 7.2 g fiber, ALM had 37.5% more fiber than CON. As almonds are rich in Mg and α-tocopherol, ALM contained 62 and 179% more than CON, respectively.

**Table 2:** Nutrient composition of control (CON) and almond (ALM) diets^a^

Nutrients	CON (*n* = 33)	ALM (*n* = 33)	*P-value* ^b^
Calories (kcal)	1643.4 ± 133.6	1665.9 ± 153.8	0.420
Crude protein (% of energy)	17.2 ± 1.6	17.5 ± 1.3	0.217
Fat (% of energy)	29.2 ± 3.1	38.4 ± 3.5	<0.001
Carbohydrate (% of energy)	55.7 ± 4.3	47.0 ± 3.4	<0.001
PUFA (g)	22.3 ± 4.5 (12%)	22.0 ± 4.1 (12%)	0.918
MUFA (g)	12.7 ± 3.3 (7%)	30.7 ± 4.0 (16%)	<0.001
SFA (g)	9.5 ± 2.6 (5%)	11.1 ± 3.3 (6%)	0.004
Dietary fiber (g)	16.0 ± 2.9	22.1 ± 3.2	<0.001
Cholesterol (mg)	125.1 ± 81.8	119.7 ± 72.2	0.696
Magnesium (mg)	209.7 ± 32.0	340.5 ± 25.1	<0.001
α-Tocopherol (mg)	7.0 ± 2.3	19.5 ± 1.7	<0.001

^a^Nutrients calculated using E-Kitchen software are presented as mean ± SD

^b^Means in the same row tested using Student’s t-test

### Anthropometry characteristics and clinical biochemistry

At the end of the 12-wk. intervention, both ALM and CON did not affect body weight, BMI, waist and hip circumferences, waist/hip ratio, % body fat, as compared to the respective baseline/washout values (Table 3). Average systolic blood pressure during the intervention was 123.6 mmHg, which is in the range of prehypertension. ALM and CON did not affect serum Mg status.

**Table 3:** Changes in anthropometric characteristics and blood biochemistries after the consumption of either control (CON) or almond (ALM) diet for 12 weeks^a^

	CON		ALM		
	Pre	Post	Pre	Post	*P-value* ^b^
Body weight (kg)	64.7 ± 10.8	64.8 ± 11.0	65.3 ± 11.0	64.7 ± 10.7	0.241
BMI (kg/m^2^)	25.3 ± 4.1	25.4 ± 4.3	25.6 ± 4.3	25.3 ± 4.2	0.148
Waist (cm)	88.1 ± 11.7	88.1 ± 12.0	89.0 ± 11.7	88.4 ± 10.7	0.477
Hip (cm)	97.5 ± 6.8	97.3 ± 7.8	97.4 ± 7.0	97.1 ± 7.4	0.467
Waist-Hip ratio	0.91 ± 0.11	0.91 ± 0.11	0.92 ± 0.11	0.91 ± 0.09	0.769
Body fat (%)	31.6 ± 8.7	31.4 ± 9.4	31.0 ± 9.0	31.7 ± 8.6	0.384
SBP (mmHg)	124.2 ± 15.2	123.6 ± 18.2	124.5 ± 14.0	122.1 ± 13.4	0.978
DBP (mmHg)	74.9 ± 10.3	77.3 ± 9.8	74.9 ± 10.6	76.5 ± 10.2	0.889
AST (U/l)	22.7 ± 11.8	22.2 ± 7.6	23.5 ± 10.1	22.4 ± 8.0	0.542
ALT (U/l)	26.1 ± 16.7	24.0 ± 11.6	26.6 ± 15.2	23.4 ± 12.7	0.822
Creatinine (mg/dl)	0.70 ± 0.14	0.71 ± 0.13	0.69 ± 0.14	0.72 ± 0.14	0.430
Mg (meq/l)	2.01 ± 0.27	2.02 ± 0.27	2.00 ± 0.27	2.03 ± 0.26	0.573

^a^Data are presented as mean ± SD

^b^P-values illustrate significant difference between post ALM and post CON values, tested using a repeated-measures ANOVA analysis, followed by LSMEANS test

### Glycemic status and lipid profile

Mean HbA1c of 2 pre-intervention values was 7.5%. At the end of ALM and CON, both diets did not change HbA1c as compared to the corresponding pre-intervention value. Similarly, both diets did not affect fasting serum glucose, insulin, and HOMA-IR values. However, as compared to the value at the end of CON, there was a trend that ALM increased C-peptide by 4.6% (*P* = 0.082). There was no difference between ALM and CON in blood glucose excursion for 3 h post breakfast, and the highest blood glucose at 246.6 and 239.4 mg/dl for ALM and CON, respectively, was noted at 90 min. The AUC of serum insulin kinetics after the breakfast did not differ between 2 diets. ALM decreased postprandial insulin level at 30, 60, and 90 min by 19, 15, and 17%, respectively, as compared to CON at the same time point. The highest postprandial blood insulin at 31.5 and 37.8 mU/l for ALM and CON, respectively, was noted at 90 min.

**Fig. 3:**
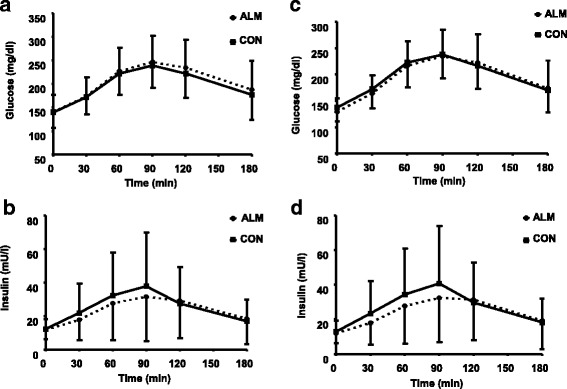
Kinetics of postprandial glucose (**a**) all subjects, *n* = 33; **c** subjects with HbA1c ≤8, *n* = 27) and insulin (**b**) all subjects; **d** subjects with HbA1c ≤8) responses to a standard breakfast after the consumption of either control (CON) or almond (ALM) diet for 12 weeks. *Means at the same time point differ, *P* ≤ 0.05, tested using a repeated-measures ANOVA analysis, followed by LSMEANS test.

Our previous data showed almonds decreased fasting blood glucose in the patients with HbA1c between 6 and 8% [15]. Thus, we conducted a subset data analysis including only subjects (*n* = 27) with the initial HbA1c ≤8% (Table 4 and Fig. 3), a level indicating a better glycemic management. Fasting serum glucose value at the end of the ALM phase was 5.9% lower than the CON phase and HbA1c value was 3.0% lower. The postprandial glycemic response to the breakfast was not different between 2 diets. Similar to the pattern noted in all 33 subjects, ALM significantly lowered the postprandial insulin level of the subset subjects at 30, 60, and 90 min by 23, 19, and 20%, respectively, as compared to CON at the same time points.

**Table 4:** Changes in serum biomarkers of glucoregulation after the consumption of either control (CON) or almond (ALM) diet for 12 weeks^a^

	CON		ALM		
	Pre	Post	Pre	Post	*P-value* ^b^
All subjects (*n* = 33)					
HbA1c (%)	7.47 ± 1.12	7.45 ± 0.80	7.51 ± 0.97	7.39 ± 1.05	0.626
Fasting plasma glucose (mg/dl)	146.5 ± 37.9	139.6 ± 33.1	140.7 ± 30.9	140.3 ± 36.8	0.587
Fasting serum insulin (mU/l)	11.2 ± 4.9	12.3 ± 6.0	10.4 ± 4.6	11.8 ± 5.7	0.782
HOMA-IR	4.00 ± 2.08	4.24 ± 2.70	3.58 ± 1.75	3.98 ± 1.98	0.833
C-peptide (ng/ml)	2.64 ± 1.12	2.60 ± 1.25	2.48 ± 0.94	2.73 ± 1.22	0.082
Postprandial glucose AUC (mg/dl∙min)	37,424 ± 7681	36,415 ± 7250	35,768 ± 7379	37,706 ± 8490	0.110
Postprandial insulin AUC (mU/l∙min)	3975 ± 2353	4690 ± 3327	4055 ± 2964	4345 ± 2791	0.557
Subjects with HbA1c ≤8 (*n* = 27)					
HbA1c (%)	7.07 ± 0.58	7.23 ± 0.63	7.18 ± 0.64	7.01 ± 0.62	0.043
Fasting serum glucose (mg/dl)	134.3 ± 24.8	137.4 ± 26.7	132.8 ± 24.8	129.3 ± 25.6	0.011
Fasting serum insulin (mU/l)	11.2 ± 5.0	12.9 ± 6.4	10.8 ± 4.8	12.0 ± 5.7	0.660
HOMA-IR	3.73 ± 2.13	4.35 ± 2.92	3.56 ± 1.80	3.83 ± 2.05	0.416
C-peptide (ng/ml)	2.62 ± 1.07	2.70 ± 1.26	2.53 ± 0.95	2.76 ± 1.23	0.351
Postprandial glucose AUC (mg/dl∙min)	34,997 ± 5591	35,921 ± 6404	34,133 ± 6477	35,637 ± 7177	0.795
Postprandial insulin AUC (mU/l∙min)	4051 ± 2424	5029 ± 3487	4284 ± 3143	4508 ± 2760	0.377

^a^Data are presented as mean ± SD

^b^P-values illustrate significant difference between post ALM and post CON values, tested using a repeated-measures ANOVA analysis, followed by LSMEANS test

Lipid profile did not differ between 2 diets in all subjects and the subset subjects (Table 5). During the whole intervention, TC and LDL-C concentration was below 200 and 105 mg/dl, respectively, and HDL-C above 40 mg/dl. There was a trend that TG level of the subset subjects was 17.9% higher at the end of ALM than CON (*P* = 0.078), but the post ALM value was more comparable to the pre ALM value with a 5.1% increase.

**Table 5:** Changes in lipid profile after the consumption of either control (CON) or almond (ALM) diet for 12 weeks^a^

	CON		ALM		
	Pre	Post	Pre	Post	*P-value* ^b^
All subjects (*n* = 33)					
TC (mg/dl)	170.7 ± 31.9	180.6 ± 36.0	175.1 ± 36.0	178.9 ± 28.4	0.468
LDL (mg/dl)	97.5 ± 29.3	102.9 ± 29.2	100.2 ± 29.5	98.0 ± 24.5	0.250
HDL (mg/dl)	44.8 ± 12.9	49.7 ± 13.4	45.2 ± 11.9	48.5 ± 12.4	0.279
TG (mg/dl)	161.1 ± 83.7	141.2 ± 68.3	164.4 ± 117.6	162.9 ± 111.1	0.183
LDL/HDL	2.34 ± 0.90	2.22 ± 0.82	2.37 ± 0.86	2.15 ± 0.70	0.485
Apo A1 (g/l)	128.9 ± 23.9	135.1 ± 23.4	128.3 ± 22.8	135.3 ± 24.3	0.776
Apo B (g/l)	82.9 ± 22.2	85.8 ± 24.2	84.3 ± 23.3	86.7 ± 22.6	0.935
Apo A1/Apo B	0.66 ± 0.20	0.66 ± 0.21	0.68 ± 0.21	0.66 ± 0.19	0.750
Subjects with HbA1c ≤8 (*n* = 27)					
TC (mg/dl)	167.7 ± 32.3	176.3 ± 32.3	172.0 ± 35.0	177.0 ± 28.4	0.735
LDL (mg/dl)	96.0 ± 29.5	103.1 ± 29.2	100.2 ± 30.7	96.8 ± 24.0	0.192
HDL (mg/dl)	45.3 ± 14.0	49.6 ± 14.0	45.5 ± 12.8	48.9 ± 13.6	0.562
TG (mg/dl)	154.3 ± 84.0	130.7 ± 54.4	146.6 ± 70.9	154.1 ± 98.6	0.078
LDL/HDL	2.30 ± 0.89	2.22 ± 0.76	2.37 ± 0.87	2.13 ± 0.70	0.327
Apo A1 (g/l)	129.2 ± 25.6	134.6 ± 25.2	128.3 ± 24.8	135.0 ± 26.6	0.675
Apo B (g/l)	80.9 ± 22.2	83.8 ± 22.3	82.5 ± 22.8	84.8 ± 22.7	0.956
Apo A1/Apo B	0.64 ± 0.19	0.64 ± 0.20	0.67 ± 0.21	0.65 ± 0.20	0.710

^a^Data are presented as mean ± SD

^b^P-values illustrate significant difference between post ALM and post CON values, tested using a repeated-measures ANOVA analysis, followed by LSMEANS test

### Inflammation, endothelial function, and oxidative stress

Serum hsCRP below 0.5 mg/dl in all 4 measurements was not affected by the diets (Table 6). While the pre-intervention NO values were comparable between the 2 diets, ALM significantly decreased serum NO by 29.8 and 29.3% in all 33 and subset subjects, respectively, as compared to CON. ALM decreased E-selectin concentration by 3.9% in all subjects (*P* = 0.121) and significantly decrease the value by 7.9% in subset subjects. Both ICAM-1 and endothelin-1 contents remained similar during the course of the trial.

**Table 6:** Changes in biomarkers of inflammation and oxidative stress after the consumption of either control (CON) or almond (ALM) diet for 12 weeks^a^

	CON		ALM		
	Pre	Post	Pre	Post	*P-value* ^b^
All subjects (*n* = 33)					
hsCRP (mg/dl)	0.20 ± 0.19	0.29 ± 0.29	0.24 ± 0.24	0.30 ± 0.29	0.796
sE-Selectin (ng/ml)	43.4 ± 20.7	44.0 ± 20.2	46.1 ± 18.0	42.3 ± 15.5	0.121
ICAM-1 (ng/ml)	223.2 ± 77.4	229.2 ± 72.2	240.1 ± 89.1	230.7 ± 80.9	0.225
Endothelin-1 (pg/ml)	0.98 ± 0.36	1.14 ± 0.41	1.04 ± 0.39	1.16 ± 0.42	0.889
Nitric oxidde (μmol/l)	59.6 ± 35.8	72.1 ± 55.3	62.5 ± 40.9	50.6 ± 26.7	0.048
Protein carbonyl (nmol/mg)	1.00 ± 0.54	1.06 ± 0.68	1.00 ± 0.65	0.72 ± 0.56	0.151
Oxidized LDL (U/l)	32.8 ± 9.9	34.8 ± 10.9	33.9 ± 10.9	35.1 ± 9.9	0.736
Urinary isoprostanes (ng/mg creatinine)	12.2 ± 6.2	14.2 ± 9.2	11.9 ± 4.2	12.2 ± 4.7	0.200
α-Tocopherol (μmol/l)	8.51 ± 3.37	9.24 ± 5.45	8.86 ± 4.43	9.57 ± 5.11	0.853
Subjects with HbA1c ≤8 (*n* = 27)					
hsCRP (mg/dl)	0.18 ± 0.19	0.29 ± 0.29	0.23 ± 0.24	0.30 ± 0.30	0.789
sE-Selectin (ng/ml)	42.3 ± 21.7	44.5 ± 21.5	45.7 ± 18.7	41.0 ± 15.4	0.026
ICAM-1 (ng/ml)	222.0 ± 78.6	229.1 ± 75.5	241.1 ± 92.8	231.5 ± 83.7	0.263
Endothelin-1 (pg/ml)	1.01 ± 0.36	1.11 ± 0.39	1.07 ± 0.38	1.16 ± 0.40	0.802
NO (μmol/l)	62.4 ± 37.1	71.2 ± 52.1	62.1 ± 41.4	50.3 ± 28.0	0.048
Protein carbonyl (nmol/mg)	1.13 ± 0.84	1.05 ± 0.67	0.96 ± 0.64	0.75 ± 0.59	0.242
Oxidized LDL (U/l)	31.5 ± 10.1	33.8 ± 10.8	32.8 ± 11.1	33.9 ± 10.2	0.653
Urinary isoprostanes (ng/mg creatinine)	12.7 ± 6.6	15.2 ± 9.8	11.7 ± 3.8	12.0 ± 4.9	0.131
α-Tocopherol (μmol/l)	8.23 ± 2.99	8.52 ± 2.83	8.25 ± 2.52	8.96 ± 3.11	0.551

^a^Data are presented as mean ± SD

^b^P-values illustrate significant difference between post ALM and post CON values, tested using a repeated-measures ANOVA analysis, followed by LSMEANS test

Circulating oxidized LDL was not affected by the diets (Table 6). ALM decreased protein carbonyl of all 33 subjects by 32% as compared to CON (*P* = 0.15). ALM did not lead to a significant increase in plasma α-tocopherol. α-Tocopherol value of all 33 subjects at the end of both ALM and CON phases was ~8% higher than the corresponding pre-intervention value, and the percent increase in the subset subjects was 8.6 and 3.5%, respectively.

## Discussion

### Glucoregulation

Approaches to protecting against CVD risk have been intensively sought for patients with T2DM. Among all evidence based approaches, a healthful dietary pattern and regular physical activity are appreciated to be the key components of diabetes management after pharmacotherapy [30]. In this study, we found that the incorporation of almonds to a healthy dietary plan might help improve long-term glycemic status in the patients with a better glucose control.

Nutrition therapy is defined as the treatment of a disease or condition through the modification of nutrient or food intake [31]. According to the nutrition therapy recommendations for the management of adults with diabetes made by the American Diabetes Association (ADA), a variety of nutrient-dense foods in appropriate portion sizes shall be incorporated into their daily diet to improve overall health and specifically to attain individualized glycemic, blood pressure, and lipid goals [31]. Both of our study diets provided about 65% of total calories from combining carbohydrate and MUFA and met the ADA recommendation that a diet have to provide 60% to 70% of calories from a mix of carbohydrate and MUFA [32]. As incorporating almonds to replace 20% energy intake shifted ~9% daily calories from carbohydrates to fats, the energy intake profile of the ALM appeared to be more consistent to the observed average macronutrient calorie distribution for the diabetes, eating ~45% of their calories from carbohydrate, ∼36–40% from fat, and ~16–18% from protein [31, 33]. As almonds are rich in oleic acid, their incorporation also raised the overall intake of MUFA, an increase considered beneficial to overall dietary fat quality [31]. HbA1c is appreciated as a biomarker for the future risks of CVD in Taiwanese [34]. Chen et al. [34] reported that patients with HbA1c ≥7.5% had 82% greater risk for CVD and 145% greater for all-cause death. Thus, it is very crucial to reduce HbA1c to the recommended level at 7%. Clinical evidence gathered from others and us [11, 12, 14, 16, 18, 35] suggested that almonds could be an integral part of a healthful diet for diabetes management through multiple mechanisms of actions, i.e., reducing glycemic index value of co-consumed food, increasing insulin secretion, and alleviating insulin resistance. Our previous study [16] showed that almonds decreased fasting blood glucose and insulin and improved HOMA-IR in Chinese patients with T2DM. In contrast, the present study did not show the same hypoglycemic effect even though the intervention duration was extended to 12 wks. These null results are added to the mixed evidence on the glycemic benefits of almonds, other individual nuts or mixed nuts. In line with our study, Lovejoy et al. [19] did not find that almonds added to either a low-fat or a high-fat diet improved glucose tolerance and insulin status in American patients with T2DM. Furthermore, Tapsell et al. [36] found that walnuts did not alter HbA1c in T2DM patients but improved fasting insulin level. While potential mechanism(s) responsible for the discrepancy remains to be explored, we speculate that better controlled lipid profile, blood glucose, oxidative stress, and inflammation noted in the study as compared to our previous trial may diminish the likelihood of detecting the benefit of almonds on glycemic control. Particularly, the baseline blood glucose and insulin values at 155.7 mg/dl and 13.7 mU/l noted in our previous study, which were larger than those in the present study, implicate that the glucose management prior to the participation in the trial may confound the potential efficacy of almonds in glucoregulation. With this notion, we did a subset analysis in patients with a better glucose management (HbA1c ≤8%) and found that almonds improved fasting blood glucose and HbA1c. Mechanism(s) by which almonds were only effective to improve glycemic management in the patients with a lower HbA1c remains to be explored, we speculate that a drug-nutrition interaction may potentially contribute to the observed divergent benefits as the patients with HbA1c >8% took more glucose lowering medications than those who had HbA1c ≤8% (2.3 ± 0.7 vs. 1.6 ± 0.8, *P* = 0.04).

### Lipid profile

Low density lipoprotein (LDL)-cholesterol levels have been shown to rise in response to the increased intake of dietary cholesterol and saturated fats [37]. Therefore, dietary counseling has become a key strategy to help manage dyslipidemia in patients with type 2 diabetes as they are at increased risk for CVD. The NCEP dietary recommendations are to consume a diet meeting the following criteria: <7% of energy from saturated fat, up to 20% of energy from MUFA, <200 mg cholesterol per day, and 25% to 35% of energy from fat [38]. The 2004 update to the NCEP guidelines can necessitate more than 50% reduction in LDL-C in some patients. Several dietary interventions have been illustrated to lower CVD risk [39, 40].

Almonds and other tree nuts have been appreciated for their hypocholesterolemic benefit, mainly due to their fiber, phytosterols, polyphenolics, and a high unsaturated to saturated fat ratio [41, 42]. The hypocholesterolemic efficacy was estimated to be 1% LDL-cholesterol reduction per 10 g almonds [13]. However, we did not find the same benefit in this study, most likely due to the baseline TC and LDL-C being below 200 mg/dL (5.172 mmol/l) and close to 100 mg/dl (2.586 mmol/l), respectively. Our results are in agreement with Ruisinger et al. study [43], in which they indicated the low baseline LDL-C [102 mg/dl (2.638 mmol/l)] value might have prohibited a significant improvement in LDL-C. Further, our results appeared consistent with the unchanged blood cholesterol levels found in our previous almond trial with the patients with coronary artery disease whose lipid profile had been well controlled either through medications, lifestyle modifications or both [44]. Thus, the cholesterol-lowering efficacy of almonds and probably other nuts becomes discernible only when total cholesterol and LDL-cholesterol level exceeds 200 and 100 mg/dl, respectively.

### Inflammation and oxidative stress

Inflammation has become one of most appreciated mechanisms accountable for the development and progression of CVD [29, 45]. Inflammatory markers, such as hsCRP, IL-6, fibrinogen, vascular cell adhesion molecule-1 (VCAM-1) and ICAM-1, have all been identified as an independent predictor for CVD or T2DM [46–51]. Sweazea et al. [52] found that almonds reduced hsCRP by ~30% in patients with T2DM, but did not change IL-6 and TNF-α. Similarly, our previous study [17] showed that almonds significantly decreased IL-6 and hsCRP. Furthermore, Rajaram et al. [53] noted almonds lowered hsCRP and E-selectin in healthy Americans. In contrast, almonds appeared ineffective to improve inflammation in the present study. These null results may be ascribed to the low inflammatory status in the study patients, evidenced by hsCRP being lower than 0.30 mg/dl, a level that is regarded with the average risk for CVD. The level of E-selectin, ICAM-1, and endothelin-1 were also in the ranges of heathy people [54, 55]. Further, as any anti-inflammatory effect of almonds may be simply secondary to the almond-mediated improvements in blood cholesterol and glucose, the unchanged inflammatory status may be a consequence of the null effects of almonds on blood cholesterol and glucose.

Nitric oxide (NO) plays a pivotal role in endothelial dysfunction and hypertension. In this study, we found that the circulating NO concentration was significantly lower at the end of ALM than CON, but it shall be noted that compared to the pre-ALM value, the post value was not significantly decreased. Given that blood pressure remained steady during the almond consumption period, clinical plausibility of the observed NO reduction in endothelial health seems not clinically important.

The unchanged plasma α-tocopherol after almond consumption was unexpected even though the dose of almonds provided 179% more α-tocopherol than the control diet. Since all subjects who completed the trial had a full compliance to the study regimen during the intervention, the unchanged circulating α-tocopherol status could be ascribed to unknown mechanism(s). Further, Traber (2014) indicated that it is very difficult to adequately interpret the circulating α-tocopherol status [56].

### Complaints and study limitation

All volunteers didn’t display any gastrointestinal symptoms and other side effects during the whole trial. There are a few limitations in this study. First, the small sample size (*n* = 33) may impose an obstacle in the detection of significant changes of the study measures. For example, we infer that a larger sample size would enhance the likelihood of detecting the benefits of almonds on LDL-cholesterol, E-selectin, protein carbonyl, and urinary isoprostanes. Second, the study was a controlled feeding trial. Thus, any potential benefits, e.g., the improved glycemic management in diabetic patients with HbA1c ≤8%, are not readily generalizable to people who add almonds to their own nutritional therapy. Third, the same batch of almonds was used in the whole study to eliminate the impact of variation in almond nutrient composition between batches on the study outcomes. However, it is unclear whether different forms of almonds would affect the study outcomes. Nevertheless, we anticipate that adding almonds to patients’ diet will definitely improve nutritional quality because of their favorable nutrient profile and potential food displacement effect.

Nutrition therapy is very crucial for managing blood glucose in patients with the diabetes. Previously, we reported that almonds added to replace 20% energy intake in Chinese patients with T2DM and mild hypercholesterolemia improved glycemic control, lipid profile, inflammation, and oxidative stress. However, the present study did not show almonds at ~60 g/d which was incorporated into the NCEP step 2 diet affected HbA1c, fasting blood glucose and insulin, postprandial glycemic response, lipid profile, and biomarkers of inflammation and oxidative stress in Chinese patients with T2DM and normocholesterolemia. In a subset analysis, the benefits of almonds in glycemic control were noted in the patients with the baseline HbA1c ≤8%.

## Conclusions

In conclusion, our findings suggest almonds incorporated to healthful diets may help improve glycemic status in diabetic patients with a better glycemic control.

## Abbreviations

ALM: Almond diet
Apo: Apolipoprotein
AUC: Area under curve
CON: Control diet
CVD: Cardiovascular disease
HDL-C: HDL-cholesterol
HOMA-IR: The homeostatic model assessment
hsCRP: High sensitivity C-reactive protein
ICAM-1: Intracellular adhesion molecule-1
LDL-C: LDL-cholesterol
MUFA: Monounsaturated fat
NCEP II: National Cholesterol Education Program step II diet
NO: Nitric oxide
PUFA: Polyunsaturated fat
SFA: Saturated fat
T2DM: Type 2 diabetes mellitus
TC: Total cholesterol
TG: Total triglycerides

## References

[1] Whorld Health. Diabetes factsheet. 2013. http://www.who.int/mediacentre/factsheets/fs312/en/.

[2] Ogurtsova K, da Rocha Fernandes JD, Huang Y, Linnenkamp U, Guariguata L, Cho NH, Cavan D, Shaw JE, Makaroff LE (2017). IDF diabetes atlas: global estimates for the prevalence of diabetes for 2015 and 2040. Diabetes Res Clin Pract.

[3] Kharroubi AT, Darwish HM (2015). Diabetes mellitus: the epidemic of the century. World J Diabetes.

[4] Srikanth S, Deedwania P (2011). Primary and secondary prevention strategy for cardiovascular disease in diabetes mellitus. Cardiol Clin.

[5] Umpierre D, Ribeiro PA, Kramer CK, Leitao CB, Zucatti AT, Azevedo MJ, Gross JL, Ribeiro JP, Schaan BD (2011). Physical activity advice only or structured exercise training and association with HbA1c levels in type 2 diabetes: a systematic review and meta-analysis. JAMA.

[6] Fox CS, Golden SH, Anderson C, Bray GA, Burke LE, de Boer IH, Deedwania P, Eckel RH, Ershow AG, Fradkin J (2015). Update on prevention of cardiovascular disease in adults with type 2 diabetes mellitus in light of recent evidence: a scientific statement from the American Heart Association and the American Diabetes Association. Diabetes Care.

[7] Evert AB, Boucher JL, Cypress M, Dunbar SA, Franz MJ, Mayer-Davis EJ, Neumiller JJ, Nwankwo R, Verdi CL, Urbanski P, Yancy WS (2014). Nutrition therapy recommendations for the management of adults with diabetes. Diabetes Care.

[8] Ley SH, Hamdy O, Mohan V, Hu FB (2014). Prevention and management of type 2 diabetes: dietary components and nutritional strategies. Lancet.

[9] Hyson DA, Schneeman BO, Davis PA (2002). Almonds and almond oil have similar effects on plasma lipids and LDL oxidation in healthy men and women. J Nutr.

[10] Jaceldo-Siegl K, Sabate J, Batech M, Fraser GE (2011). Influence of body mass index and serum lipids on the cholesterol-lowering effects of almonds in free-living individuals. Nutr Metab Cardiovasc Dis.

[11] Jenkins DJ, Kendall CW, Josse AR, Salvatore S, Brighenti F, Augustin LS, Ellis PR, Vidgen E, Rao AV (2006). Almonds decrease postprandial glycemia, insulinemia, and oxidative damage in healthy individuals. J Nutr.

[12] Jenkins DJ, Kendall CW, Marchie A, Josse AR, Nguyen TH, Faulkner DA, Lapsley KG, Singer W (2008). Effect of almonds on insulin secretion and insulin resistance in nondiabetic hyperlipidemic subjects: a randomized controlled crossover trial. Metabolism.

[13] Jenkins DJ, Kendall CW, Marchie A, Parker TL, Connelly PW, Qian W, Haight JS, Faulkner D, Vidgen E, Lapsley KG, Spiller GA (2002). Dose response of almonds on coronary heart disease risk factors: blood lipids, oxidized low-density lipoproteins, lipoprotein (a), homocysteine, and pulmonary nitric oxide: a randomized, controlled, crossover trial. Circulation.

[14] Josse AR, Kendall CW, Augustin LS, Ellis PR, Jenkins DJ (2007). Almonds and postprandial glycemia--a dose-response study. Metabolism.

[15] Li N, Jia X, Chen CY, Blumberg JB, Song Y, Zhang W, Zhang X, Ma G, Chen J (2007). Almond consumption reduces oxidative DNA damage and lipid peroxidation in male smokers. J Nutr.

[16] Li SC, Liu YH, Liu JF, Chang WH, Chen CM, Chen CY (2011). Almond consumption improved glycemic control and lipid profiles in patients with type 2 diabetes mellitus. Metabolism.

[17] Liu JF, Liu YH, Chen CM, Chang WH, Chen CY (2013). The effect of almonds on inflammation and oxidative stress in Chinese patients with type 2 diabetes mellitus: a randomized crossover controlled feeding trial. Eur J Nutr.

[18] Wien M, Bleich D, Raghuwanshi M, Gould-Forgerite S, Gomes J, Monahan-Couch L, Oda K (2010). Almond consumption and cardiovascular risk factors in adults with prediabetes. J Am Coll Nutr.

[19] Lovejoy JC, Most MM, Lefevre M, Greenway FL, Rood JC (2002). Effect of diets enriched in almonds on insulin action and serum lipids in adults with normal glucose tolerance or type 2 diabetes. Am J Clin Nutr.

[20] National Cholesterol Education Program (NCEP) Expert Panel on Detection E, and Treatment of High Blood Cholesterol in Adults (Adult Treatment Panel III) (2002). Third report of the National Cholesterol Education Program (NCEP) expert panel on detection, evaluation, and treatment of high blood cholesterol in adults (adult treatment panel III) final report. Circulation.

[21] Guerre-Millo M (2008). Adiponectin: an update. Diabetes Metab.

[22] Heinonen MV, Laaksonen DE, Karhu T, Karhunen L, Laitinen T, Kainulainen S, Rissanen A, Niskanen L, Herzig KH (2009). Effect of diet-induced weight loss on plasma apelin and cytokine levels in individuals with the metabolic syndrome. Nutr Metab Cardiovasc Dis.

[23] Lihn AS, Pedersen SB, Richelsen B (2005). Adiponectin: action, regulation and association to insulin sensitivity. Obes Rev.

[24] Tan VM, Wu T, Henry CJ, Lee YS (2015). Glycaemic and insulin responses, glycaemic index and insulinaemic index values of rice between three Asian ethnic groups. Br J Nutr.

[25] Thomas M, Massa G, Bourguignon JP, Craen M, De Schepper J, de Zegher F, Dooms L, Du Caju M, Francois I, Heinrichs C (2001). Final height in children with idiopathic growth hormone deficiency treated with recombinant human growth hormone: the Belgian experience. Horm Res.

[26] Laporte M, Villalon L, Thibodeau J, Payette H (2001). Validity and reliability of simple nutrition screening tools adapted to the elderly population in healthcare facilities. J Nutr Health Aging.

[27] Walter MF, Blumberg JB, Dolnikowski GG, Handelman GJ (2000). Streamlined F2-isoprostane analysis in plasma and urine with high-performance liquid chromatography and gas chromatography/mass spectroscopy. Anal Biochem.

[28] Bieri JG, Tolliver TJ, Catignani GL (1979). Simultaneous determination of alpha-tocopherol and retinol in plasma or red cells by high pressure liquid chromatography. Am J Clin Nutr.

[29] Danesh J, Wheeler JG, Hirschfield GM, Eda S, Eiriksdottir G, Rumley A, Lowe GDO, Pepys MB, Gudnason V (2004). C-reactive protein and other circulating markers of inflammation in the prediction of coronary heart disease. N Engl J Med.

[30] Franz MJ, Boucher JL, Evert AB (2014). Evidence-based diabetes nutrition therapy recommendations are effective: the key is individualization. Diabetes Metab Syndr Obes.

[31] Evert AB, Boucher JL, Cypress M, Dunbar SA, Franz MJ, Mayer-Davis EJ, Neumiller JJ, Nwankwo R, Verdi CL, Urbanski P, Yancy WS (2013). Nutrition therapy recommendations for the management of adults with diabetes. Diabetes Care.

[32] Association AD (2015). (4) foundations of care: education, nutrition, physical activity, smoking cessation, psychosocial care, and immunization. Diabetes Care.

[33] Oza-Frank R, Cheng YJ, Narayan KM, Gregg EW (2009). Trends in nutrient intake among adults with diabetes in the United States: 1988-2004. J Am Diet Assoc.

[34] Chen YY, Lin YJ, Chong E, Chen PC, Chao TF, Chen SA, Chien KL (2015). The impact of diabetes mellitus and corresponding HbA1c levels on the future risks of cardiovascular disease and mortality: a representative cohort study in Taiwan. PLoS One.

[35] Cohen AE, Johnston CS (2011). Almond ingestion at mealtime reduces postprandial glycemia and chronic ingestion reduces hemoglobin a (1c) in individuals with well-controlled type 2 diabetes mellitus. Metabolism.

[36] Tapsell LC, Batterham MJ, Teuss G, Tan SY, Dalton S, Quick CJ, Gillen LJ, Charlton KE (2009). Long-term effects of increased dietary polyunsaturated fat from walnuts on metabolic parameters in type II diabetes. Eur J Clin Nutr.

[37] Siri-Tarino PW, Sun Q, Hu FB, Krauss RM (2010). Saturated fatty acids and risk of coronary heart disease: modulation by replacement nutrients. Curr Atheroscler Rep.

[38] Pasternak RC. Report of the Adult Treatment Panel III: the 2001 National Cholesterol Education Program guidelines on the detection, evaluation and treatment of elevated cholesterol in adults. Cardiol Clin. 2003, 21:393–8. 10.1016/s0733-8651(03)00080-8 14621453

[39] Konig D, Bonner G (2007). Berg a: [the role of adiposity and inactivity in primary prevention of cardiovascular disease]. Herz.

[40] Lichtenstein AH, Ausman LM, Jalbert SM, Vilella-Bach M, Jauhiainen M, McGladdery S, Erkkila AT, Ehnholm C, Frohlich J, Schaefer EJ (2002). Efficacy of a therapeutic lifestyle change/step 2 diet in moderately hypercholesterolemic middle-aged and elderly female and male subjects. J Lipid Res.

[41] Chen CY, Lapsley K, Blumberg J (2006). A nutrition and health perspective on almonds. J Sci Food Agric.

[42] Chen C, Blumberg JB (2008). Phytochemical composition of nuts. Asia Pac J Clin Nutr.

[43] Ruisinger JF, Gibson CA, Backes JM, Smith BK, Sullivan DK, Moriarty PM, Kris-Etherton P (2015). Statins and almonds to lower lipoproteins (the STALL study). J Clin Lipidol.

[44] Chen CY, Holbrook M, Duess MA, Dohadwala MM, Hamburg NM, Asztalos BF, Milbury PE, Blumberg JB, Vita JA (2015). Effect of almond consumption on vascular function in patients with coronary artery disease: a randomized, controlled, cross-over trial. Nutr J.

[45] Festa A, D'Agostino R, Tracy RP, Haffner SM, Study IRA (2002). Elevated levels of acute-phase proteins and plasminogen activator inhibitor-1 predict the development of type 2 diabetes: the insulin resistance atherosclerosis study. Diabetes.

[46] Pradhan AD, Manson JE, Rifai N, Buring JE, Ridker PM (2001). C-reactive protein, interleukin 6, and risk of developing type 2 diabetes mellitus. JAMA.

[47] Pradhan AD, Ridker PM (2002). Do atherosclerosis and type 2 diabetes share a common inflammatory basis?. Eur Heart J.

[48] Luc G, Bard J-M, Juhan-Vague I, Ferrieres J, Evans A, Amouyel P, Arveiler D, Fruchart J-C, Ducimetiere P, Group PS (2003). C-reactive protein, interleukin-6, and fibrinogen as predictors of coronary heart disease: the PRIME study. Arterioscler Thromb Vasc Biol.

[49] Asegaonkar SB, Marathe A, Tekade ML, Cherekar L, Bavikar J, Bardapurkar J, Ajay R (2011). High-sensitivity C-reactive protein: a novel cardiovascular risk predictor in type 2 diabetics with normal lipid profile. J Diabetes Complicat.

[50] Soinio M, Marniemi J, Laakso M, Lehto S, Rönnemaa T (2006). High-sensitivity C-reactive protein and coronary heart disease mortality in patients with type 2 diabetes: a 7-year follow-up study. Diabetes Care.

[51] Zhang H, Park Y, Wu J, Chen X, Lee S, Yang J, Dellsperger KC, Zhang C (2009). Role of TNF-alpha in vascular dysfunction. Clin Sci (Lond).

[52] Sweazea KL, Johnston CS, Ricklefs KD, Petersen KN (2014). Almond supplementation in the absence of dietary advice significantly reduces C-reactive protein in subjects with type 2 diabetes. J Funct Foods.

[53] Rajaram S, Connell KM, Sabate J (2010). Effect of almond-enriched high-monounsaturated fat diet on selected markers of inflammation: a randomised, controlled, crossover study. Br J Nutr.

[54] Glowinska B, Urban M, Peczynska J, Florys B (2005). Soluble adhesion molecules (sICAM-1, sVCAM-1) and selectins (sE selectin, sP selectin, sL selectin) levels in children and adolescents with obesity, hypertension, and diabetes. Metabolism.

[55] Nash MC, Wade AM, Shah V, Dillon MJ (1996). Normal levels of soluble E-selectin, soluble intercellular adhesion molecule-1 (sICAM-1), and soluble vascular cell adhesion molecule-1 (sVCAM-1) decrease with age. Clin Exp Immunol.

[56] Traber MG (2014). Vitamin E inadequacy in humans: causes and consequences. Adv Nutr.

